# 

**Published:** 1852-12

**Authors:** 


### CONTENTS

OF THE

### MEDICAL EXAMINER.

NEW SERIES—NO. XCVI.—DECEMBER, 1852.

### ORIGINAL COMMUNICATIONS.

Surgical Sketches. By W. E. Horner, M. D., Prof. of Anatomy in the University of Pennsylvania, Senior Surgeon at the St. Joseph's Hospital, &c. &c. A Military Hospital at Buffalo, New York, in the year 1814, - - - - - 753

A History of the Epidemic Cholera, which prevailed, during the summer of 1852, at Chambersburg, Pa. By A. H. Senseny, M. D., - - - - - - - 774

Adhesive Plaster, as a valuable Constituent in the composition of various apparatus used in Surgery. By D. Gilbert, M. D., Professor of Surgery in the Medical Department of Pennsylvania College, - - - - - - 784

Review of M. Bernard's Theory of an Hepatico-Renal Circulation. By D. B. Phillips, M. D., Assistant Surgeon, U. S. N., and Member of the Academy of Natural Sciences of Philadelphia, 787

### CLINICAL REPORTS.

Pennsylvania College, Ninth below Locust street. Service of Professor Gilbert. Reported by W. H. Gobrecht, M. D., - 790

### BIBLIOGRAPHICAL NOTICES.

The Prescriber's Complete Handbook, comprising the principles of the art of Prescribing, &c. By MM. Trousseau and Reveil. Edited, with notes, by J. Birkbeck Nevins, M. D., - - 802

A Practical Treatise on Dental Medicine, being a Compendium of Medical Science, as connected with the study of Dental Surgery, &c. &c. Second Edition. Revised, corrected and enlarged by Thos. E. Bond, A. M., M. D., Professor of Special Pathology, &c. &c. in Baltimore Dental College, - - 805

### EDITORIAL.

Medical Department U. S. Navy, - - - - - 808

Death of Dr. Jonathan Cowdery, - - - - - 808

Death of Dr. Daniel Drake, - - - - - 808

Dr. Brown-Séquard, - - - - - - 808

